# Parasite load effects on sex ratio, size, survival and mating fitness of *Heleidomermis magnapapula* in *Culicoides sonorensis*

**DOI:** 10.2478/jofnem-2023-0052

**Published:** 2023-11-23

**Authors:** Bradley A. Mullens, Katherine A. Luhring

**Affiliations:** Department of Entomology, University of California, Riverside, California 92521, USA; Present Address: 2291 SW 2^nd^ Avenue, Fruitland, Idaho 83619

**Keywords:** Mermithidae, Diptera, Ceratopogonidae, *Culicoides*, biological control

## Abstract

*Heleidomermis magnapapula* parasitizes the blood-feeding midge *Culicoides sonorensis*. Most (84%) single mermithid infective second stage juveniles (J2) developed into adult females, while parasitism by multiple J2 yielded 97% male adults. Nematodes emerged from the midge larval host as adults and mated immediately; females were ovoviviparous. Host larvae were exposed to nematode J2 and examined intact microscopically to score initial parasite load. Midge hosts were reared individually. Premature midge death, nematode survival within the host, and emerging adult nematode sex ratio and size as a function of load and host size were all tracked. Higher nematode loads produced smaller adult nematode males. The higher loads also increased and accelerated premature host death. Emergence of > 7–9 adult nematode males was rare, but up to 19 tiny males emerged from a single host. Larger midges supported higher parasite loads and a larger total volume of emerged nematode biomass. Virgin adult nematode males then were paired with females of variable, known sizes (volume) and held to determine size effects on fertility (egg hatch), and male survival (longevity). Tested adult males ranged in size from 0.0025 – 0.0334 mm^3^ and females from 0.0121 – 0.1110 mm^3^. Logistic regression indicated female nematode fertility was positively influenced by male nematode size, while nematode load and female nematode size had no significant effect. While fertility was reduced statistically in smaller males, even some of the smallest male and female individuals could be fertile. Findings are related to field studies in this system.

Mermithid nematodes are common parasites of arthropods, especially insects, and thus have been studied as potential biological control agents for pest insects damaging crop production or human and animal health (Poinar, 1979; Petersen, 1985; Platzer, 2007). An important general feature of mermithid biology involves density-dependent sex determination of the parasitic nematodes, as observed nearly a century ago by Christie (1929). Superparasitism of hosts (multiple parasites attacking and entering the same host) by mermithids is common, and a greater number of nematode parasites within a host yields a higher proportion of males (e.g., Petersen, 1972; Sanad et al., 2013).

This provides a powerful population regulation feedback mechanism that operates on multiple levels. The proportion of females produced during periods of higher nematode parasite activity (and thus more parasites per host) tends to be smaller, yielding a relatively stable level of host exploitation over time (Tingley and Anderson, 1986; Blackmore and Charnov, 1989). Superparasitism reduces available host resources per parasite, leading to the emergence of both more males and proportionally fewer and smaller nematode females that may be less fecund (Blackmore and Charnov, 1989). Host resources, in turn, also are affected by variation in suitability of the host environment, indicated by factors such as food availability (Petersen, 1972; Craig and Webster, 1982).

*Heleidomermis magnapapula* (Mermithida: Mermithidae) is an important parasite of the blood-feeding midge *Culicoides sonorensis* (Diptera: Ceratopogonidae), a key vector of bluetongue and related viruses in domestic and wild ruminants in North America (Poinar and Mullens, 1987; Purse et al, 2015). The North American species *H. magnapapula* is by far the best-known member of the genus, but other mermithids attack biting midges across much of the globe (Mullens et al., 2008).

Several mermithid species that attack mosquitoes (Diptera: Culicidae) have been colonized and tested for biological control of mosquitoes in aquatic habitats (see Petersen, 1985; Platzer, 2007). The primary mosquito-infecting mermithids that have been studied, notably *Romanomermis culicivorax*, *R. iyengari* and *Strelkovimermis spiculatus*, are typical mermithids in that they emerge as later-stage juveniles from the insect host. Once in the environment, the mermithids molt to adults, mate, and lay eggs in the benthic soil at the bottom of a shallow water body. Eggs can survive for weeks or perhaps months, and provide a resistant life stage, allowing them to be transported and distributed for biological control in the field (Petersen, 1985; Platzer, 2007).

In contrast to the mosquito-infecting mermithids, *H. magnapapula* emerges from the insect midge as an adult. After adult mating, the eggs hatch internally (ovoviparously) and the adult female nematode disperses the mobile second-stage juveniles (J2) into the environment (Mullens and Velten, 1994; Mullens and Luhring, 1998). This allows the mermithid to match the rapid development of *C. sonorensis* in often-ephemeral wet mud habitats, such as dairy wastewater ponds or water trough overflow (Mullens and Velten, 1994; Mullens and Luhring, 1998). Although *H. magnapapula* lacks a resistant life stage that might aid in practical biological control, surveys have shown that *H. magnapapula* can cause high levels of mortality in *C. sonorensis* in the field, sometimes exceeding 60% (Paine and Mullens, 1994; Mullens and Luhring, 1998). Thus, *H. magnapapula* is regarded as the most important known natural enemy of *C. sonorensis*, and it is important to understand the relationship between the nematode parasite and its host (Mullens and Luhring, 1998).

Diet generally impacts the size and fecundity that individual insects of a particular species can attain (see Beukeboom, 2018), and host nutrition in turn can affect mermithid development inside those hosts (Petersen, 1972; Jiao et al, 2016). In mosquitoes, higher loads of mermithids result in lower proportional nematode parasite emergence from the mosquito host, higher premature host mortality, and/or slower host development (Petersen, 1972; Petersen and Willis, 1972; Petersen, 1978; Galloway and Brust, 1985; Sanad et al., 2017). The size of *C. sonorensis* also is greatly impacted by organic input (which likely affects insect nutrition) into its developmental sites in the field (Mullens and Rodriguez 1988). The size of the insect host is thus an important consideration for studying mermithid-host interactions.

The mermithid *H. magnapapula* is known to adjust its sex ratios in response to parasite load. Single nematode parasites in a host can develop into females or occasionally relatively large males, while more than two parasites in a host develop into males (Mullens and Velten, 1994; Paine and Mullens, 1994; Mullens and Luhring, 1996; Mullens and Luhring, 1998). As discussed above, host size (and nutritional status) might interact with mermithid development as well. In the present study, we examine that relationship in more detail, determining the extent to which superparasitism impacts insect host (and thus nematode parasite) survival and results in adult nematodes of different sizes, possibly influencing their subsequent fitness and survival outside the insect host.

## Materials and Methods

### Routine Culture

**Figure 1:**
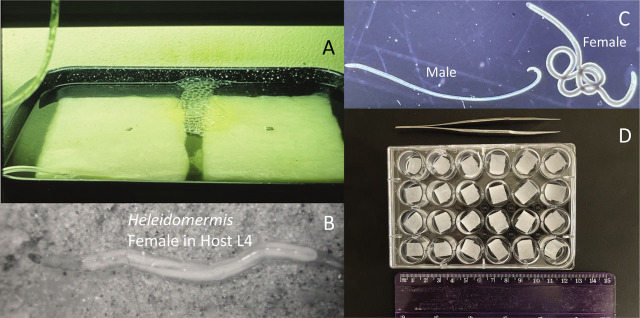
A. Host midge rearing pan used for colony maintenance of both *Culicoides sonorensis* (midge host) and its mermithid parasite, *Heleidomermis magnapapula*. B. Mermithid adult female about to emerge from its 4^th^ larval instar host (L4). C. Adult male (left) and female (right) of *H. magnapapula* (photo by Jack Kelly Clark, University of California, Davis). D. 24-well plastic tray for rearing individual, parasitized midges.

The *C. sonorensis* were a colony (AA strain) kindly provided by the USDA Arthropod-Borne Animal Diseases Laboratory in Laramie, Wyoming. The *H. magnapapula* were started by nematodes emerging from naturally parasitized *C. sonorensis* that were collected from dairy wastewater ponds in western Riverside County, California (Mullens and Velten, 1994). For routine maintenance, *H. magnapapula* was maintained in its midge host, *C. sonorensis*. The colonies were reared at 28 °C at a 13L:11D photoperiod in enamel pans (42-cm-long, 28-cm-wide and 5-cm-deep) of nutrient-rich water in the laboratory (Fig. 1A) (Mullens and Velten, 1994; Luhring and Mullens, 1997). The pans contained two completely saturated polyester batting pads (3-cm thick) with room around the pads’ perimeter for water circulation stimulated by air bubblers. Pads extended to the water surface, were densely fibrous, and provided a substrate for a rich microbial community (especially bacteria, algae, and fungi) to provide food for the host larvae.

*Culicoides sonorensis* has four larval instars (L1, L2, L3 and L4). Host larvae were exposed to the nematodes at 7 d (late L2 and early L3) by placing 16 fully gravid female mermithids (just beginning to produce live J2) onto the batting surface in each pan. This resulted in approximately 14,400 J2 to attack the 3,000–4,000 host larvae/pan (Luhring and Mullens, 1997). The late L4 hosts were sieved from the pans 5 to 9 days later and were held in 9-cm glass Petri dishes of dechlorinated tap water for adult nematode emergence. While the L4 larvae were in the Petri dishes, the bacterial-feeding nematode *Panagrellus redivivus* was offered as food (Mullens and Velten, 1994). Adult mermithids, particularly females, sometimes could be seen even with the naked eye through the midge host cuticle before emergence (Fig. 1B). After nematode emergence, nematodes were separated from remaining L4 hosts using an 80-mesh sieve, which only the nematodes could crawl through. We also reared *C. sonorensis* in non-infected pans using the same larval rearing methods, but with no exposure to mermithids.

### Tracking Parasite Load and Survival of *H. magnapapula*

A small-scale version of the above rearing conditions was used. Six-day-old *C. sonorensis* larvae (late L2 and early L3) were harvested from the routine non-infected rearing pans using a fine mesh sieve. Approximately 250 host larvae were placed into each of several 9-cm glass Petri dishes containing a disc of polyester batting and 50 ml of nutrient-rich rearing pan water from non-infested host pans. A single fully gravid *H. magnapapula* female (just beginning to produce live J2) was added to a dish. Based on her length, which is positively correlated with fecundity (Mullens and Velten, 1994), the number of J2 she held could be estimated: in general, an average female produced about 900 J2 (Luhring and Mullens, 1997). This yielded an average parasite load of about 3.5 nematodes per midge host and about 75% parasitism (Luhring and Mullens, 1997). The range of parasite loads was substantial, however, and allowed us to select midge hosts with a large range of numbers of J2, as detailed below.

After exposure to the J2 for approximately 24 h, hosts again were sieved from the Petri dishes. Individual host midge larvae were placed on a microscope slide in a drop of dechlorinated water and a cover slip was placed on top. The amount of water was adjusted as needed. A tissue was used to wick water from under the cover slip or a pipette was used to add small amounts of water to the cover slip edge. This method, after some practice, could gently press the midge larva between the slide and cover slip, restricting its movements without causing injury. The live host larva on the slide was placed under a phase contrast microscope and examined at 40–100×, where J2 could be seen through the translucent host cuticle (Mullens and Luhring, 1997). This allowed us to generate hosts with known nematode parasite loads for further study. The sex of host *Culicoides* larvae cannot presently be determined morphologically.

Midge larvae with known nematode loads, including unparasitized controls, were reared individually in 24-well plastic sorting trays with 16-mm-diam wells (Fig. 1D). Each well had a piece of folded filter paper, and nutrient-rich pan water (as described above) was added and then replaced every 2–3 days from uninfested routine rearing pans. The filter paper provided a substrate for the larva to occupy, a site for pupation, and also served to support growth of microorganisms to mimic the conditions of the regular rearing pans.

Wells were checked daily to see if both the host larva and any unemerged nematodes still inside died, if midges pupated, or if adult nematode parasites emerged (which kills the host insect). If adult nematode parasites successfully emerged, their number and sex were recorded. If the host midge larvae died prior to emergence of parasites, the hosts were dissected to determine the number and sex (if sufficiently developed) of unemerged mermithids inside the host body. The dissections were done by first cutting off the midge larva's head at the base of the head capsule. The midge body contents then were extruded in a drop of water on a glass microscope slide by gently holding the posterior tip of the host body using fine forceps, and then using a second pair of forceps to compress the body partially, sliding the second forceps toward the anterior. Adult male and female nematodes are easily differentiated: males are thinner, shorter and more translucent, lack a vagina, and have a distinctly curved caudal tip and spicules (Poinar and Mullens, 1987) (Fig. 1C). Nematodes and body contents easily came out through the open anterior end of the body cavity.

### Determining Male Nematode Parasite Volume Relative to Midge Parasite Load

Host midge larvae (L4) that had been exposed in the routine parasite production pans were harvested as usual (5–9 days after exposure to J2). Those hosts were then held individually in dechlorinated tap water in 96-well plastic ELISA plates with 7-mm-diam wells. They were checked daily until host death, pupation, or nematode parasite emergence. Number and sex of emerging nematodes were recorded for each midge host yielding parasites. After adult nematode emergence (and invariably the death of the host), remains of the host midge larva and emerged nematodes were placed into hot (60 to 70 °C) water. These measures denatured proteins and helped prevent any changes in nematode shape, while causing them to straighten out, making measurements (using an ocular micrometer) easier. Nematodes and midge host remains were placed into a vial with 2% formalin and 3% glycerin, which kept them in good condition until measurements could be taken. These were done using a wet mount on a glass slide, with specimens under a cover slip and fluid level maintained to avoid deforming the specimen. The host larval head capsule was then measured at its widest point. Nematode width for males was measured at two points: the narrowest at the posterior end near the spicule and the widest at the widest point near the anterior end. Females were measured at the posterior end of the ovary and at the widest point in the middle, posterior to the vagina.

The two width measurements were averaged to yield an estimate of width (diameter), and the volume of the nematode thus was estimated using the formula for the volume of a cylinder (π r^2^h). If more than five males emerged from a single host, five representative males were selected and measured for the data set. Formal randomized choice of the selected males was not done, but males from the same host were generally the same size and similar in appearance.

### Determining Male Nematode Mating Fitness as a Function of Nematode Volume

Parasitized L4 hosts were held individually, as described above, in wells of a 96-well plastic plate, and were checked daily for mermithid emergence. In the rare cases where both sexes emerged from the same host, all of those nematodes were excluded from mating experiments. The nematodes used for these mating fitness studies thus were virgins, having had no prior access to the opposite sex.

Each nematode was then placed together with one member of the opposite sex in a well of a 24- well sorting tray (16-mm-diam wells). Males were always less than 24 h old when paired with a potential mate. Virgin females were less abundant, and thus were up to 48 h old when paired. Upon introduction to the wells, the sexes were gently moved into contact with each other using a small metal dental pick, to encourage mating. Typically, the nematodes immediately coiled around each other as soon as they made contact. Following being placed together, the nematodes were held together at room temperature (24 °C). Survival and egg hatch of nematodes were checked daily (sometimes only one day on weekends) for seven days. Presence of J2 (evidence of successful insemination) was recorded; the typical period for *H. magnapapula* embryogenesis at that temperature is about 3.5 days (Mullens et al., 1995). Dead nematodes were recorded as well; female nematodes typically die within 1–2 days of giving birth if inseminated (Mullens and Velten, 1994). Seven days after pairing, nematodes were killed in hot water and preserved. Both males and females were measured for length and average width, as described above.

## Results

### Time of Premature Midge Host Death as a Function of Nematode Parasite Load

If *H. magnapapula* emerge successfully, they begin to do so at 10 to 11 days after infection (Mullens and Velten, 1994). Table 1 shows the number and time of death of parasitized hosts (and unparasitized controls) that died without either the midge host pupating or adult parasites emerging. This is termed premature death, in which both the insect and nematodes inside it die. A weighted mean time of death was calculated first for each parasite load category. The weighted means were not analyzed statistically, but provided a useful overview of when premature death occurred.

**Table 1:** Time of premature host death (death of host and unemerged parasites) over time as a function of parasite load for *Culicoides sonorensis* host larvae exposed on day 0 to infective-stage immatures (J2) of *Heleidomermis magnapapula*. Parasites counted after dissection of dead host.

**Parasite Load (N)**	**Time of Death (Days after Parasitism)**				

	**1–3**	**4–6**	**7–9**	**10+**	**Mean (Range)**
0 (5)	1	1	0	3	10.6 (2–21)
1 (24)	8	2	5	9	8.8 (1–28)
2–3 (27)	10	2	6	8	6.4 (1–21)
4–5 (17)	11	2	1	3	4.6 (1–16)
6–7 (12)	6	2	1	3	5.6 (1–15)
8–9 (9)	7	1	1	0	2.9 (1–8)
10 + (17)	8	6	1	2	4.2 (1–13)

Only five unparasitized host larvae (controls) died prematurely, with a weighted mean time of death of 10.6 days after the J2 exposures in infected hosts. This was too few to analyze statistically. Among parasitized midge hosts, the time of premature death tended to decrease with parasite load and was tested using chi-square (χ^2^) analysis. Based on our observations on numbers of mermithids emerging from parasitized midges collected in the past in the field (Paine and Mullens, 1994; Mullens and Luhring, 1998), a load of 1 was common and considered “light”, a load of 2 to 3 was also common and was considered “moderate”, and a load of 4+ was uncommon and considered “heavy.”

In order to place sufficient numbers of premature deaths in categories to facilitate χ^2^ analysis (traditionally ≥ 5/cell) (McDonald, 2014), deaths were pooled into load categories of 1, 2 to 3, and 4+, that were matched against four time categories in a 3 × 4 contingency table. The χ^2^ value was 13.84 (*p* < 0.05). So, as parasite loads for parasitized hosts increased, time of premature host death decreased significantly.

### Fate of Parasitized Hosts as a Function of Parasite Load

**Figure 2:**
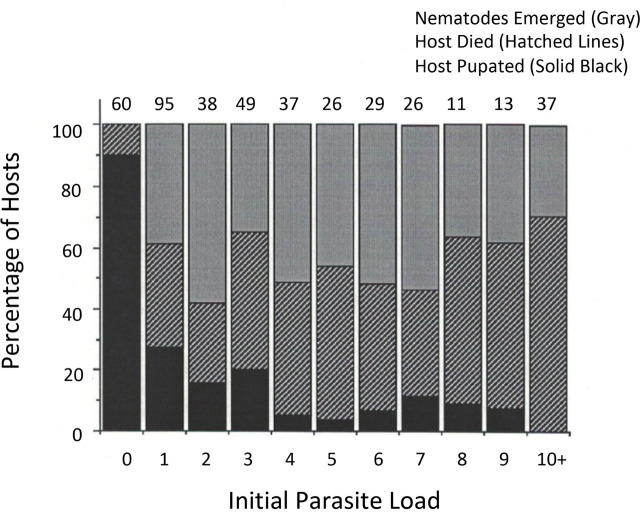
Patterns of successful nematode emergence, premature host death (death of host and parasites inside), and successful host pupation as they varied with *Heleidomermis magnapapula* initial parasite load. Number of J2 per host was determined by microscopic examination of *Culicoides sonorensis* larval hosts 24 h after exposure. The number of hosts reared for that parasite load is shown above each histogram bar.

Fig. 2 shows the fate of midge hosts and nematodes, based on the visual assessment of how many J2 had initially penetrated the late L2 or early L3 host larva. Control midges had 90% pupation and 10% died before pupation. In marked contrast, 12.2% of parasitized midges (all loads) pupated and 87.8% of parasitized hosts (all loads) died, either prematurely or when nematodes emerged successfully from the host larva. The cell frequencies (parasitized vs control, pupated or died) were compared using a 2 × 2 χ^2^ test. Parasitism reduced host survival very significantly χ^2^ = 156.07, *p* < 0.001).

Among parasitized midge larvae, in which at least one J2 was observed immediately after exposure, linear regression analysis was conducted to test whether parasite load (as the independent variable) influenced each of the three parameters of interest as a dependent variable (i.e., whether the slope of the regression differed from zero). Sample sizes per parasite load category ranged from 11 to 95 (Fig. 2). In the first regression, the percentage of midges yielding some nematodes that emerged successfully from the larval host ranged from 57.9% (load = 2) to 29.7% (load = 10+) and was not influenced by parasite load (*t* = −1.17, *p* = 0.28).

The other two dependent variables were affected substantially by parasite load. The percentage of midges pupating was reduced at higher nematode parasite loads (*t* = 4.83, *p* < 0.01). For hosts where a single J2 was observed immediately after exposure, 27.4% pupated. In general, for loads of ≥ 4, pupation was below 11.5%, and no hosts with ≥ 10 parasites survived to pupate. The percentage of hosts dying prematurely, without nematodes emerging, increased with parasite load (*t* = 3.51, *p* < 0.01). For loads ≥ 8, premature death occurred in the majority of hosts (Fig. 2).

Comparing visual estimates of parasite load with midge dissection data, the initial visual assessments of J2 per host usually were the same or more than subsequent nematode counts derived from midge dissection after death or live nematode emergence (in 92% of cases). Some larval hosts, however, yielded slightly more parasites than had been seen initially through the host cuticle by visual microscopic observation. That is, we had not seen every J2, and the visual estimates had been slightly conservative. In 13 of 158 determinations (8%), more nematodes were present by emergence or dissection than had been counted by visual examination through the host cuticle immediately after exposure. This source of error (undercounting) was evenly scattered through the range of nematode parasite loads (five cases with loads of 1 to 3, four cases with loads of 4 to 6, and four cases with loads > 7). The visual estimates were accurate predictors of dissected J2 numbers overall, and results were very similar (compare Table 1 using dissection of dead hosts and Fig. 2 using visual load estimates).

**Figure 3:**
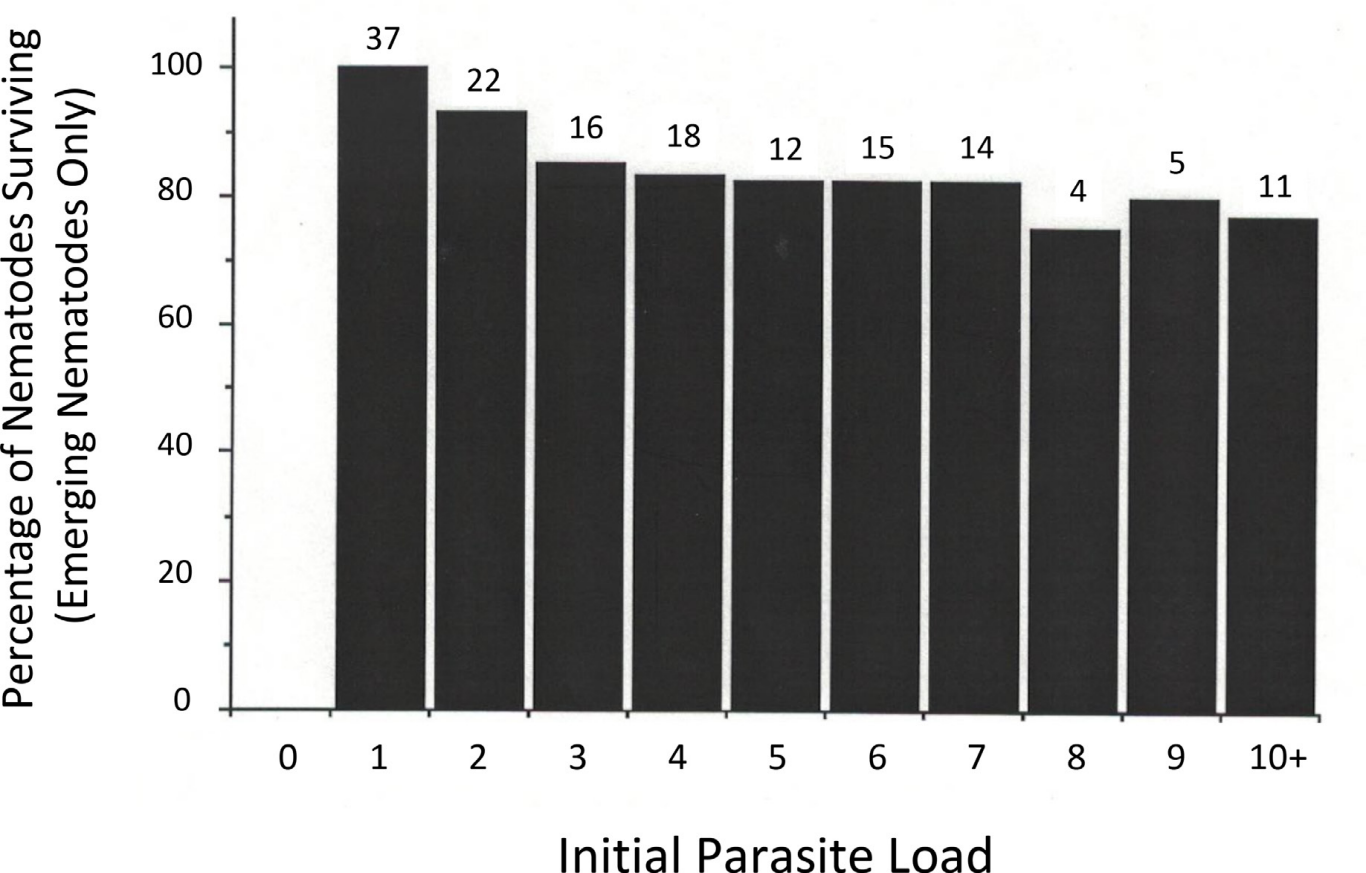
Survival percentage (proportion emerging) of multiple *Heleidomermis magnapapula* in *Culicoides sonorensis* host larvae as a function of initial parasite load (*n* noted above histogram bars). Load estimates were based on microscopic examination of live hosts 24 h after exposure to J2 using one nematode per host as a baseline, and then rearing individual hosts. Only hosts from which live mermithid adults emerged were used in this calculation.

Looking more closely only at midge hosts from which live nematodes emerged, it was possible to compare initial visual load estimates with the number of live parasites that emerged for each parasite load level. This provides an estimate of nematode survival as a function of load (Fig. 3). In this calculation, for midges containing a single nematode (J2 counted 24 h after host exposure), successful emergence constituted 100% survival, although even single parasites caused some increase in premature host mortality (Fig. 2). For hosts that were initially judged to contain multiple nematode parasites, nematode parasite survival estimates ranged from 93% (for an estimated initial load of two) to 75 to 80% (for initial loads ≥ 8). Thus, there appeared to be a slight decline in survival as a function of initial nematode density.

To test this, where we had adequate numbers of mermithids for analysis, a χ^2^ analysis (Table 2) was performed to examine number of nematodes alive versus number missing and presumed dead in designated host-load categories. Categories were pooled to yield a better sample size (> 10 in this case) per group (Table 2). For hosts harboring multiple J2 initially, load did not significantly impact eventual successful emergence of adult *H. magnapapula* (*p* > 0.4).

**Table 2:** Survival of *Heleidomermis magnapapula* preparasites in host *Culicoides sonorensis* larvae (successful live adult nematode emergence) as a function of initial parasite load (number of preparasites seen within each host). Only hosts producing some live adult parasites included.

**Initial Load**	**# Live Adults**	**# Dead (Missing)**
2–3	82	10
4–5	132	23
6–7	154	34
8–9	72	16

Chi-square (3 df) = 2.89, *p* = 0.409

### Effect of Initial Parasite Load on Sex Ratio of Emerging Nematodes

When midge hosts were penetrated by a single J2 (load observed immediately after exposure) that emerged successfully, 83.7% were female (*n* = 31 out of 37) and 16.3% (6 out of 37) were male. For individual loads of 2 to 9 nematodes per host, the large majority (97% of hosts) yielded exclusively males.

The few exceptions are listed as follows: 1) one midge host with an initial load of two yielded a single female nematode (one died sometime during development); 2) one midge host with an initial load of three yielded one male and one female (and one died); and 3) one host with an initial load of six nematodes yielded two males and one female (and three nematodes died). In subsequent fertility testing, only single females emerging from a host, or males emerging with other males from a host (known virgins), were used.

### Effect of Variation in Midge Host Size on Nematode Development and Emergence

**Figure 4:**
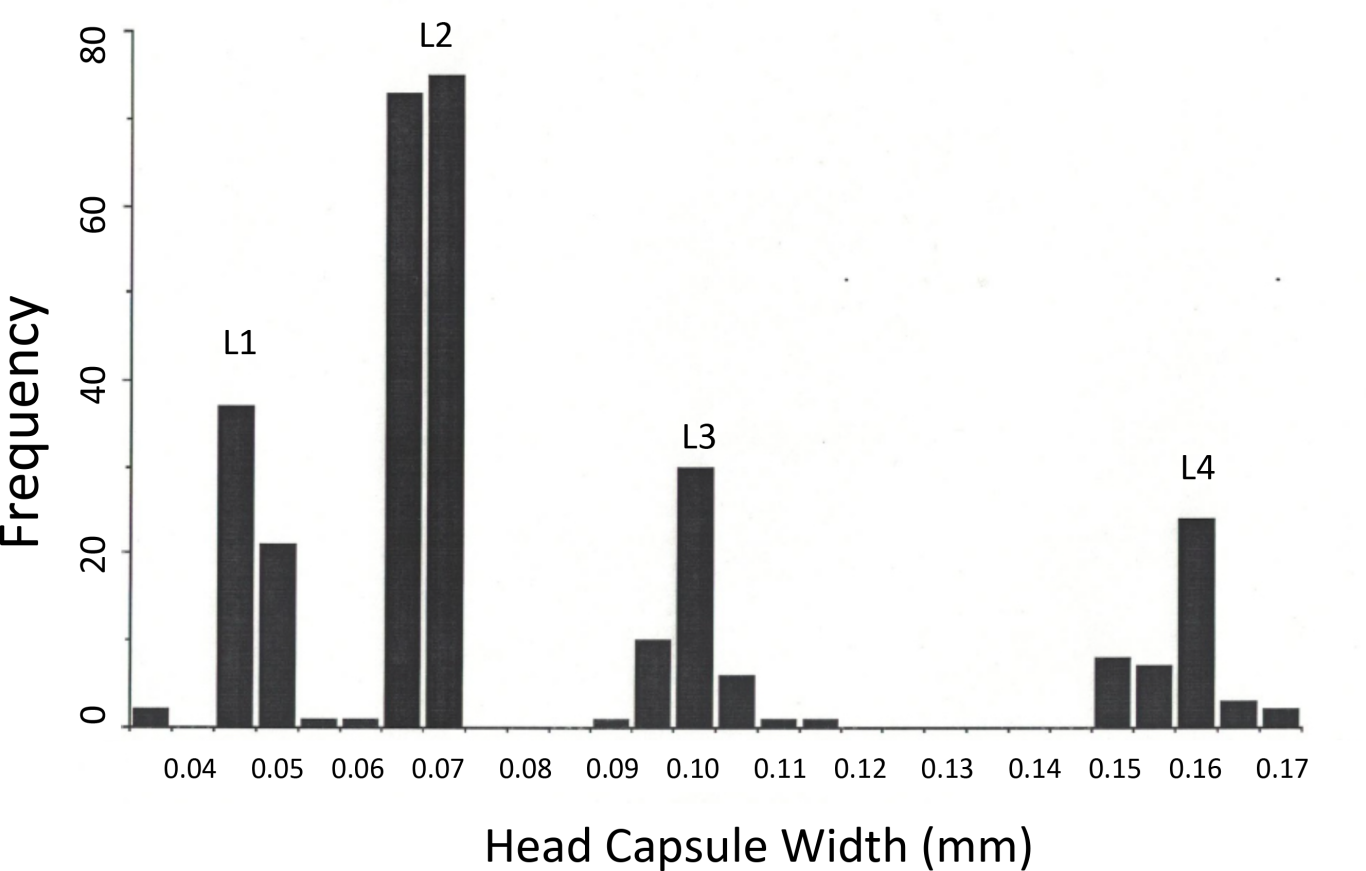
Frequency distribution of larval head capsule widths for the UCR colony of *Culicoides sonorensis*. Four host larval instars (L1 to L4) indicated.

One possible influence on nematode development and survival is variation in host size. In the present system, our host size measure was larval head capsule width near the base. Head width for the four host larval instars, taken from uninfected UCR colony material, is shown in Fig. 4. Following Dyar's law (Dyar, 1890), dimensions of relatively sclerotized body parts (such as head capsules) in insects usually separate into discrete groupings by instar, and this is true for larvae of *C. sonorensis*, including the UCR colony (Barnard and Jones, 1980; Abubekerov and Mullens, 2018). The L4 head capsule sizes typically ranged from about 150–170 μ.

Midge host larvae cannot be sexed based on external morphology, but the pupae can (Shults et al., 2016). Based on head capsule width measurements from cast larval skins and differences in the shape of the tip of the resulting pupal abdomen between males and females, female midge hosts (*n* = 50) had average L4 head widths of 164 ± 1 μ, while males (*n* = 40) had average L4 head widths of 161 ± 2 μ and did not differ significantly using a two-sample *t* test (*t* = 1.19; *p* > 0.2). It was not possible to successfully separate the host sexes based on larval head capsule width.

**Figure 5:**
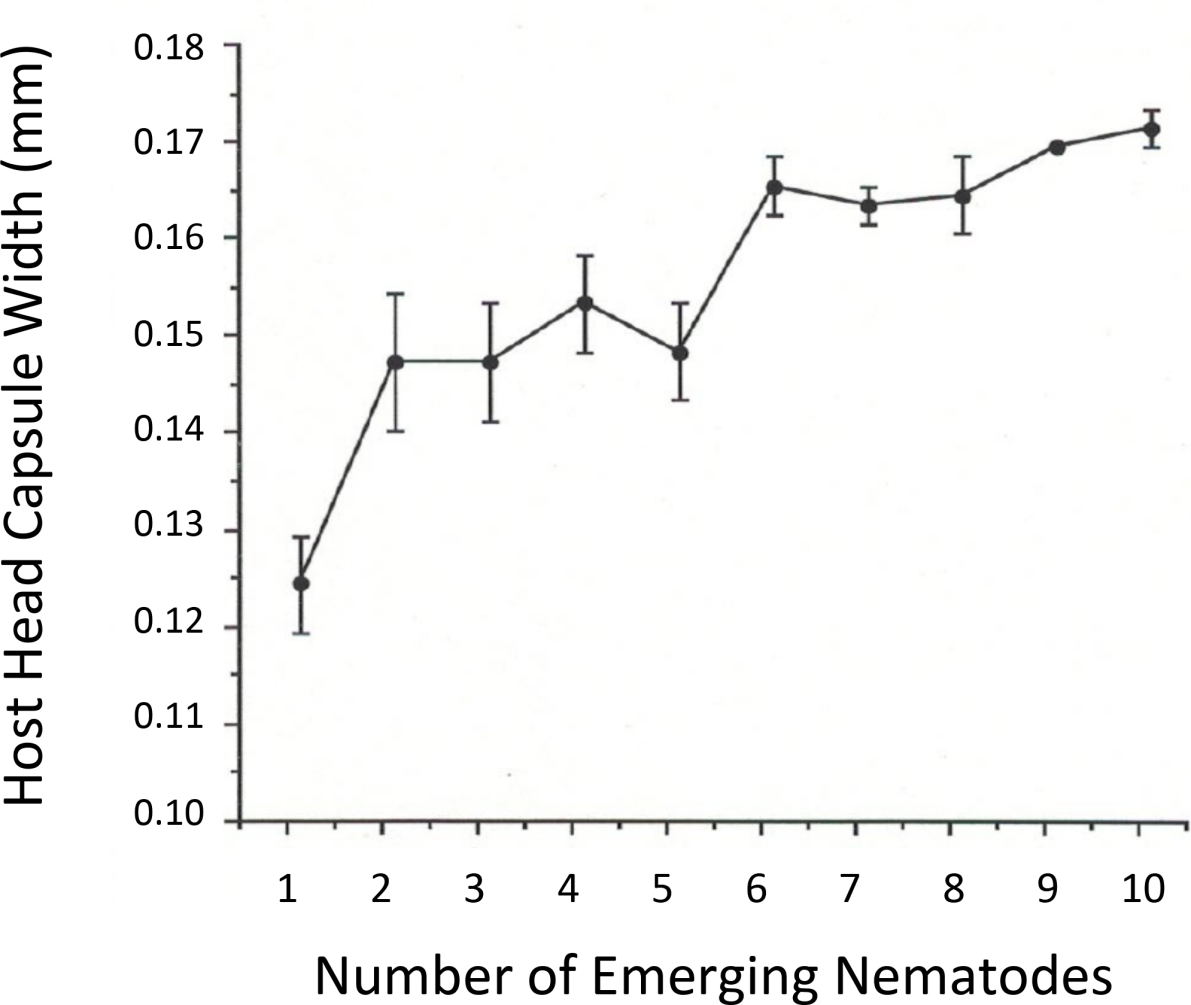
Average (± sd) host size (L4 head capsule width in mm) plotted against the number of male *Heleidomermis magnapapula* produced from individual host larvae of *Culicoides sonorensis*.

Examining a plot of midge host size (L4 head width) versus nematode parasite load, host size did appear to have a positive influence on *H. magnapapula* across the range of parasite loads. Hosts yielding a single live adult *H. magnapapula* averaged about 125 μ in head width, while the few hosts yielding 9–10 mermithid adults averaged about 170 μ (Fig. 5).

### Effect of Parasite Load on Male Nematode Size and Total Volume per Host

**Figure 6:**
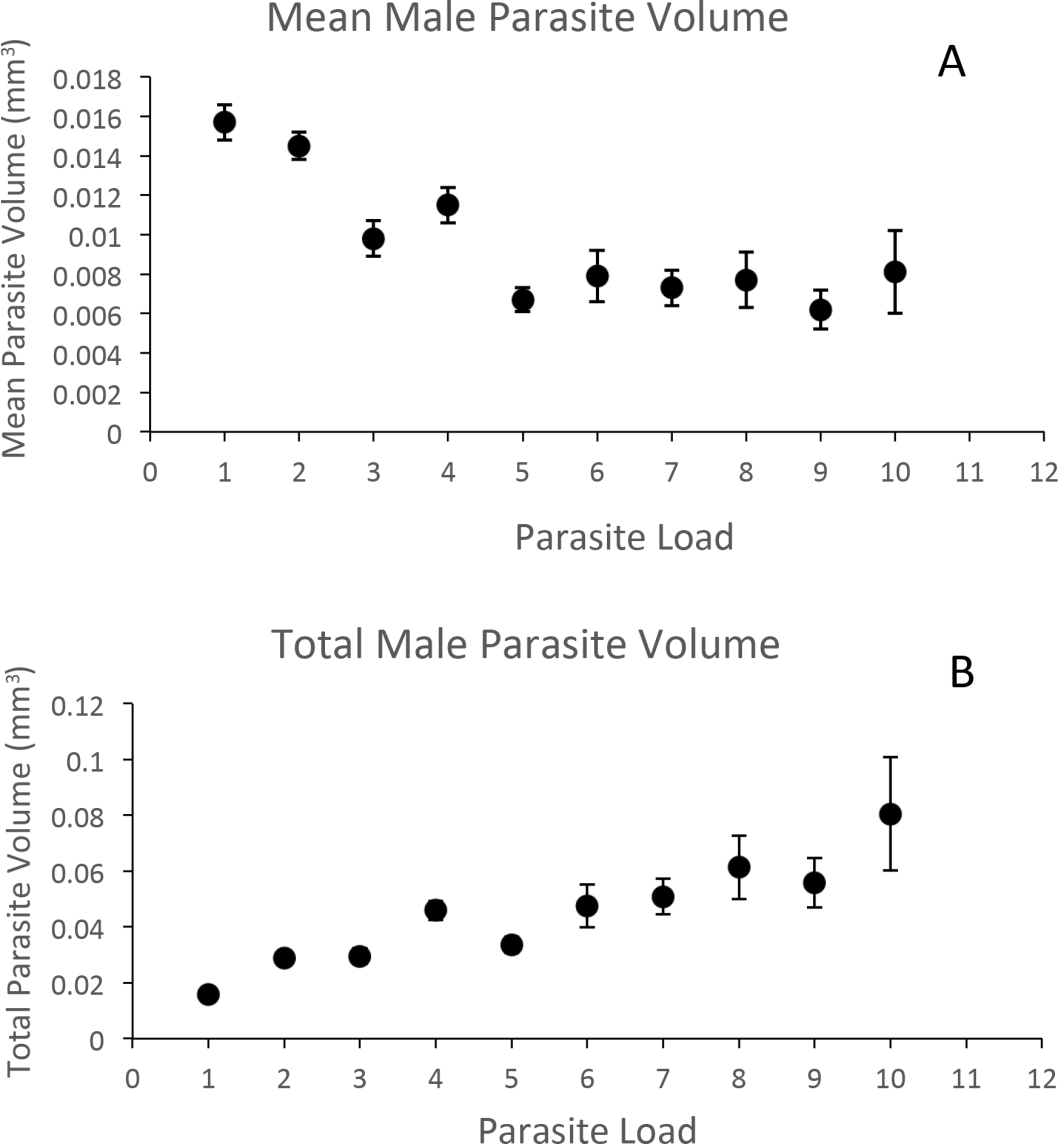
A. Average (± sd) volume (cubic mm) of individual male *Heleidomermis magnapapula* emerging from individual host larvae of *Culicoides sonorensis* harboring different numbers of emerged male nematodes. B. Average (± sd) sum of male volume for *Heleidomermis magnapapula* emerging from individual host larvae of *Culicoides sonorensis* harboring different numbers of emerged male nematodes.

Increasing parasite loads had a substantial negative impact on average size of the emerging males (Fig. 6A). For midge hosts yielding a single live nematode male, those males averaged about 0.016 mm^3^ in volume. In contrast, average male volume was less than half of this number (< 0.008 mm^3^) for hosts yielding ≥ 7 males. A multiple regression was conducted with average individual male volume as the dependent variable, and the independent variables of host size (head capsule width) and the parasite load as independent variables. This was done for the entire range of parasite loads observed. In a general linear model (GLM), the most important factor influencing average individual male volume was load (a negative influence) (*F* = 8.89; *p* < 0.0001), followed by host size (a positive influence) (*F* = 10.61; *p* < 0.01), and the interaction of load × size was not significant (*F* = 1.68; *p* > 0.1).

The increase of nematode parasite loads varied with total volume of nematodes emerging per midge host (Fig. 6B). It is important to keep in mind that it was the larger host larvae that tended to support successful emergence of larger numbers of nematodes (Fig. 5). That said, single successful male emergences yielded a nematode volume of 0.02 mm^3^, but ≥ 7 males per host, while smaller as individuals, produced a cumulative nematode volume of > 0.05 mm^3^ per host. A multiple regression was conducted with total male volume as the dependent variable, with independent variables of host size (head capsule width) and parasite load. This was done for the entire range of parasite loads observed. In this generalized linear model (GLM), the positive effect of load was most significant (*F* = 23.6; *p* < 0.0001), followed by a very significant positive effect of host size (*F* = 13.45; *p* < 0.001), and the load × size interaction (*F* = 3.49; *p* < 0.01).

### Nematode Size Relationships with Mating Success and Male Survival

**Figure 7:**
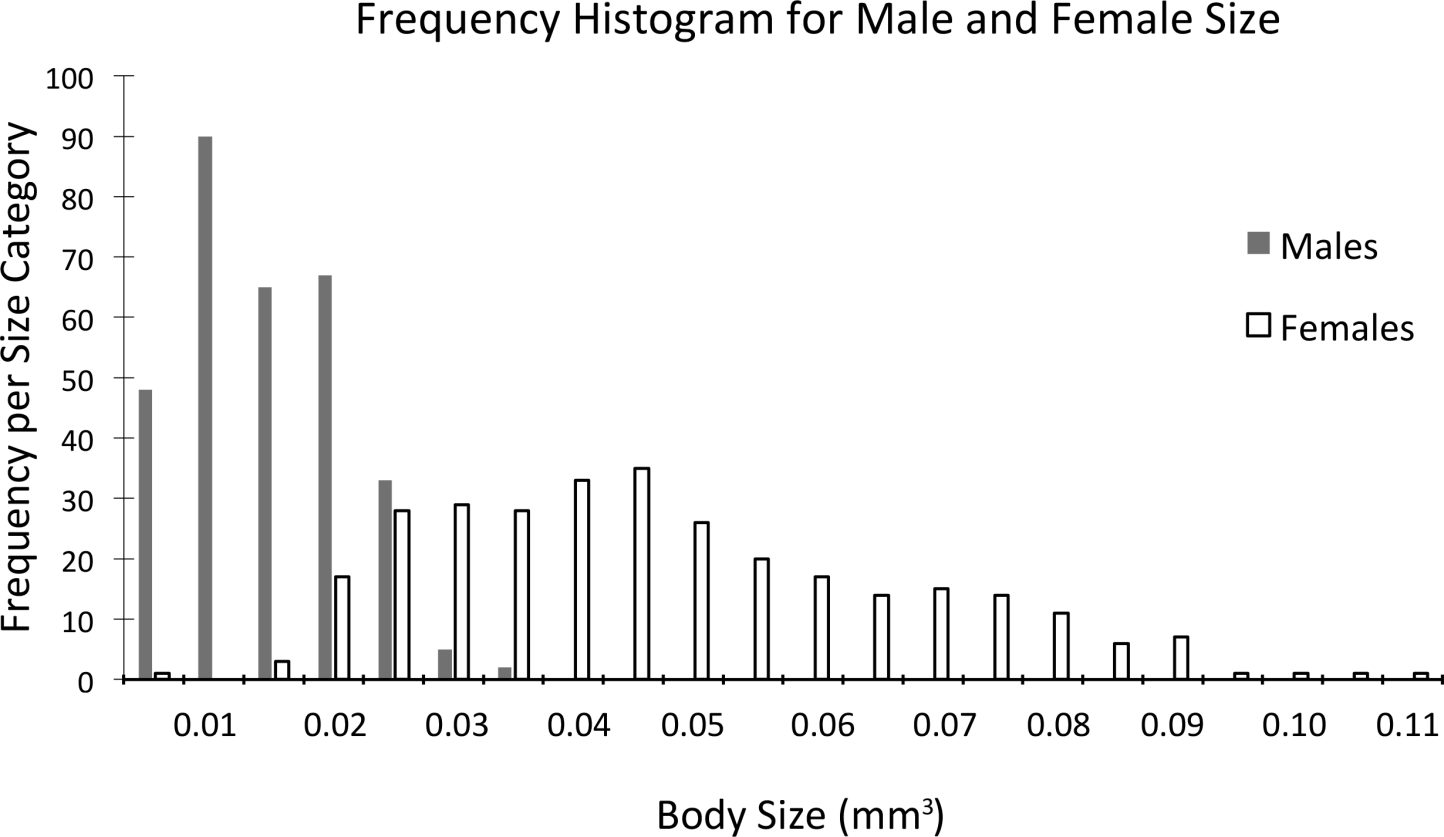
Frequency distribution of emerged adult male and female *Heleidomermis magnapapula* used in tests of size versus fertility (virgin pairs).

The frequency distribution for the range of nematode sizes used in the individual mating pair experiment is shown in Fig. 7. Males ranged in size from 0.0025 to 0.0334 mm^3^ (a 13.4 × range). Females ranged in size from 0.0121 to 0.1110 mm^3^ (a 9.2 × range) (Table 3).

**Table 3:** Size (as estimated by volume, in mm^3^) categories of emerged *Heleidomermis magnapapula* used in logistic regression analyses of survival and mating success.

**Sex**	**Category**	**Mean mm^3^ (SE)**	**Min.**	**Max.**	**N**
Male	1	0.0041 (0.0001)	0.0025	0.0054	58
	2	0.0078 (0.0002)	0.0056	0.0104	87
	3	0.0127 (0.0002)	0.0106	0.0154	60
	4	0.0179 (0.0002)	0.0155	0.0202	64
	5	0.0240 (0.0005)	0.0206	0.0334	37
Female	1	0.0204 (0.0004)	0.0121	0.0254	49
	2	0.0376 (0.0006)	0.0261	0.0504	151
	3	0.0627 (0.0008)	0.0506	0.0754	81
	4	0.0896 (0.0019)	0.0757	0.1110	27

SE = standard error.

Over all ranges of respective sizes in the pairing of individual virgin males and individual virgin females, 118 of 313 pairs (37.7%) resulted in insemination, as assessed by egg hatch. Internal hatching of eggs within the nematode female's body was almost always either 0% or 100%. There were two exceptions: one individual female had about 25% of the egg complement hatch, and another had about 75% of its complement hatch. Thus, 116 out of 118 (98.3%) inseminated females had all their eggs hatch.

A preliminary Kruskal-Wallis test was utilized first as a rough indicator of the effect of size on fertility. This was done by dividing the male or female nematodes into two groups based on whether their eggs hatched or not. Males that successfully mated had a 35% larger median volume (0.0131 mm^3^) versus males that had not mated successfully (0.0097 mm^3^) (*H* = 15.11, df =1, *p* < 0.001). Fertile females, in contrast, actually were 12% smaller (median 0.0398 mm^3^) than infertile females (median 0.0451 mm^3^) (*H* = 4.22, df = 1, *p* = 0.04). However, even the very smallest nematode individuals of each sex were among the pairs that were fertile, so there seemed to be no strict lower limit on mating success. Looking separately and across the range of mate sizes at the 50 smallest nematode males (< 0.005 mm), nine mated successfully (18%). For the 50 smallest nematode females (< 0.025 mm^3^), 18 mated successfully (36%). The smallest fertile male was 0.0037 mm^3^, while the smallest fertile female was 0.0147 mm^3^.

But this separate analysis of the sexes did not account for the likely simultaneous and interacting influence of the size of the mate on fertility. For this more comprehensive analysis, the *H. magnapapula* were sorted into size classes based on volume, with five categories for males and four for females (Table 3).

Logistic regression analysis was conducted, with female fertility (positive or negative) as a variable dependent on male and female size categories (volume) and the number of males per host. Results are shown in Table 4. Parameter estimates for female nematode volume (χ^2^ = −0.005; *p* > 0.1) and number of male nematodes per host (χ^2^ = −0.008; *p* > 0.1) were negative, but were not statistically significant. On the other hand, male nematode size (volume) had a very large positive effect on female nematode fertility (χ^2^ = 0.034; *p* < 0.001). Larger nematode males were much better able to inseminate females, while all sizes of females were still able to be inseminated.

**Table 4:** Results of logistic regression testing the effects of *Heleidomermis magnapapula* male volume (MVolume), *H. magnapapula* female volume FVolume), and number of males per host (Nummale) on fertility (egg hatch) of previously virgin mating pairs.

**Variable**	**Estimate (SE)**	**Wald χ^2^**	**P**	**Stnd Estimate**
Intercpt	−0.833 (0.408)	4.166	0.041	
MVolume	0.034 (0.010)	12.112	0.0005	0.245
FVolume	−0.005 (0.003)	2.354	0.125	−0.108
Nummale	−0.008 (0.006)	2.125	0.145	−0.101

**Figure 8:**
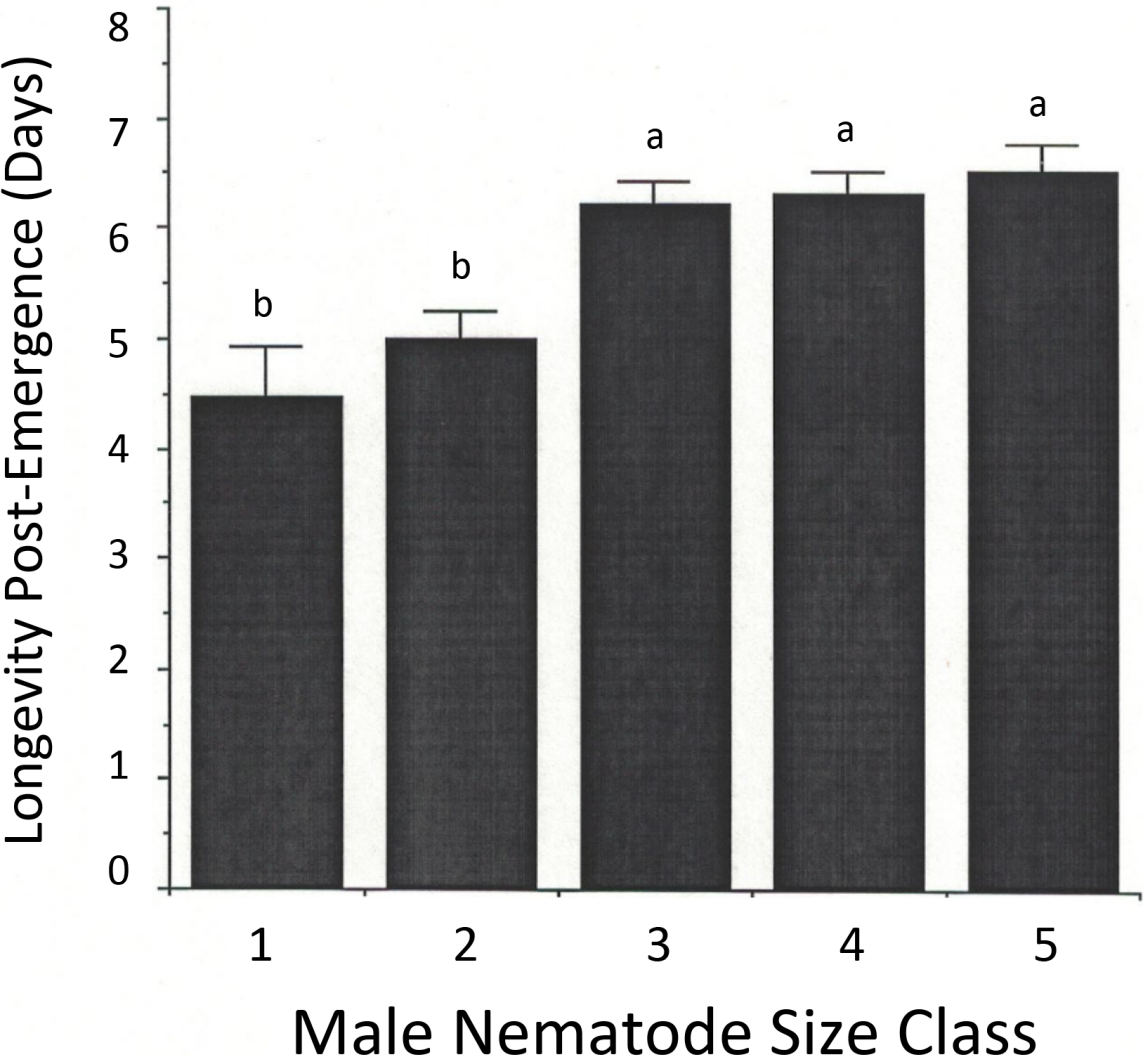
Average (± sd) survival of different size categories of male *Heleidomermis magnapapula* (see Table 3) in water after emerging from host larvae of *Culicoides sonorensis*. Different letters above histogram bars indicate statistically significant differences (*p* < 0.05).

Male nematode survival in water (all pairs) is shown in Fig. 8. A GLM analysis (ANOVA) utilized the five male size categories as the independent variable and the number of days each survived as the dependent variable. Large males lived longer (*F* = 9.85; *p* < 0.001). The smaller males (size categories 1 and 2) lived an average of about 4.5 to 5 days, while larger males (size categories 3 to 5) lived an average of 6 to 6.5 days. Female nematode survival was not assessed, since females die about 3.5 days after mating, after producing J2 (Mullens and Velten, 1994). Unmated females can survive in water up to two weeks at 23 °C (Mullens and Velten, 1994), but our study did not follow females for that long.

## Discussion

This study examined factors influencing nematode and host midge development and subsequent nematode fitness (post-emergence) under extreme manipulation of initial nematode parasite load in the laboratory. The unusual biological features of *H. magnapapula* include emergence from the host as an adult in 1.5 to 2 weeks after host entry (214 degree-days above 9 °C), immediate mating, and internal egg hatch and placement by adult females of J2 mermithids in close proximity to host larvae in the field (just above waterline) after only a few days of embryogenesis (44 degree-days above 10 °C) (Mullens and Velten, 1994; Mullens et al., 1995; Mullens and Luhring 1998). Together these biological characteristics lead to the nematode's ability to complete a summer life cycle in only about 2.5 to 3 weeks. This is an adaptation to the equally rapid life cycle of its host midge, *C. sonorensis* (Mullens and Lii, 1987).

The habitats (shallow, silty mud in ponded or slow-moving water, usually polluted by animal excrement) used by *C. sonorensis* and *H. magnapapula* vary immensely over both temporal and spatial scales. They are often highly ephemeral, patchy and unpredictable.

While the best literature comparisons for this study are studies about mermithids attacking mosquito larvae, it is good to keep in mind the fundamentally different biology of the more famous mosquito mermithids such as *R. culicivorax*, *R. iyengari* or *S. spiculatus*, which have more typical mermithid life histories (Petersen, 1985; Platzer, 2007; Sanad et al., 2017). For example, *H. magnapapula* does not need to molt to the adult stage after host emergence and before mating, or lay eggs in habitat substrates that may persist for weeks or more before hatch. Indeed, it was the unusually rapid life cycle of *H. magnapapula* that allowed us to expeditiously assess aspects such as survival, embryogenesis, or mating fitness of emerged nematode adults as a function of variation in individual nematode parasite load and midge host size.

The ability to gauge nematode parasite loads visually through the nearly transparent cuticle, without harming the host, has been described and used before by Blackmore (1992) for *Romanomermis* in several mosquito species, and by Mullens and Luhring (1998) for *H. magnapapula*. The technique has also been used extensively and productively by Sanad et al. (2017) for detailed studies of *R. iyengari* and *S. spiculatus* in *Culex quinquefasciatus*. Despite occasional undercounting in the present study, this was a critically useful technique, providing a baseline estimate of initial nematode load while then allowing those individual hosts to be reared throughout the period of mermithid development. The Sanad et al. (2017) study exposed hosts to individual J2, and then further exposed those hosts as desired to precisely adjusted load in small (0.1 ml) drops of water. Plain water (no substrate) is not a natural environment for *H. magnapapula* or *C. sonorensis*. The present study exposed groups of hosts to groups of J2 in a rearing substrate, only a short time later assessing loads visually. It is very unusual to see successful *H. magnapapula* emergence of more than 7 to 9 per host, but one case of 19 emerging males was documented here.

In the laboratory, very high numbers of attacking *H. magnapapula* J2 will immobilize and kill hosts quickly; Mullens and Luhring (1998) observed as many as 28 J2 within a single dead midge larva exposed in a dish of water, but this was an unusual case. Interestingly, the mosquito mermithids *R. iyengari* and *S. spiculatus* recognize previously parasitized hosts and prefer hosts that are either unparasitized or have lower loads (Sanad et al., 2017). The present methods did not allow detection of such an effect in *H. magnapapula*, if it exists.

Of midge hosts that died prematurely in the present study, and based on dissection of those dead hosts, high loads of more than eight per host resulted in hosts dying after only 3 to 4 days, while hosts with single parasites, if they died prematurely, died after about 9 days. Based on visual load assessment immediately after J2 exposure, hosts in the laboratory were surprisingly resilient in tolerating even rather high parasitism, and some nematodes usually could emerge from superparasitized hosts. However, loads ≥ 8 killed hosts prematurely well over half of the time, with subsequent death also of the mermithids within.

Excessively high loads of *H. magnapapula* are maladaptive and are not very common in nature. In the wild, based on successful emergence of *H. magnapapula* from mature L4 host larvae, loads of ≤ 2 were observed in a limited sampling of *C*. *variipennis* in New York State, but parasitism was low overall (averaging 7% at one site and 10% at another) and the male:female sex ratio was 0.7 (Mullens and Rutz, 1982). In more intensive surveys in California in the closely related host *C. sonorensis*, and with somewhat higher levels of parasitism (12% overall), the sex ratio of successfully emerging adult nematodes was 2.4, with a maximum of 8 nematode males emerging from a naturally-parasitized, field-collected midge host (Paine and Mullens 1994). Based on dissection of usually immature nematodes from the four larval instars of *C. sonorensis* collected in the field, loads averaged 1.7 to 2.9 per host, and point prevalence nematode parasite loads ≥ 3 (larvae from a particular collection) were seen in ≤ 26% of midge larvae in any instar (Mullens and Luhring 1998). Average parasite loads of 2 to 3 are probably optimal for this nematode.

The general decline in *H. magnapapula* survival and size at higher loads was accompanied by evidence that larger hosts are more likely to support higher loads and, in fact, larger hosts supported a higher mermithid biomass. This likely reflects competition for host resources and raises the possible influence of host diet, which has been well documented in other mermithid systems. Well-fed host *C. quinquefasciatus* larvae containing a single nematode produced 13% males, while starved hosts with single parasites produced 92% males (Petersen 1972). Craig and Webster (1982) even surgically transferred partially-developed immature *Mermis nigrescens* to a second grasshopper host, allowing the parasite to grow larger than those that remained in the original host.

Jiao et al. (2016) showed that the sex ratio of *Ovimermis sinensis* in the moth *Helicoverpa armigera* was related to several host and parasite factors such as load, but ultimately could be modified by manipulating host diet. The mosquito *Aedes vexans* (Meigen) parasitized by *R. culicivorax* showed not only premature host mortality, but also reduced size, delayed molting, and loss of fat body relative to unparasitized mosquitoes (Galloway and Brust, 1985). Blackmore (1992) also used nematode volume as a surrogate for mass in *Romanomermis* sp. attacking several different mosquito species. In those field studies, total parasite volume was independent of load, but individual parasites became smaller with higher loads, again suggesting competition for host resources. The present studies with *H. magnapapula* were done in the laboratory, used experimental exposures of young hosts (L2 to L3) over a broad range of nematode parasite loads, and featured full rearing studies, which probably imposed a larger range of stressors and consequent variation than typically would be seen in the field.

An unusual aspect of the present study was an examination of the impact of the size of adult *H. magnapapula* on mating success. This was made possible specifically by the extreme range of adult nematode sizes that could be generated experimentally in the laboratory by manipulating parasite load. Larger adult female *H. magnapapula* are more fecund, ranging from a J2 yield of about 500 for a 6-mm-long female to 2500 in a 13-mm long-female (Mullens and Velten, 1994). In the present study, male size significantly impacted fertility (mating success), while female size did not. Even the smallest individual male and female nematodes could achieve some (albeit perhaps low) level of mating success; we were not able to demonstrate a threshold size below which mating was not possible. Further, if eggs hatched at all, even the smallest individuals typically displayed close to 100% egg hatch, meaning there was little or no indication of sperm limitation.

There was also some tendency (not statistically significant in the present analyses) for larger females to be less fertile in the single pair matings. Further study might be useful to investigate whether this reflects female mate choice (rejecting small males in certain pairs), or if mating is not physically possible between a very large female and a very small male. The overall fertility for these individual pair experiments (37%) was relatively lower than the 53 to 63% experimental mating success of 1 to 2 females paired with 1 to 2 males of typical colony size (Luhring and Mullens 1997). And, while it has not been carefully documented, it has been rare to find females in the *H. magnapapula* colony that had no egg hatch (Mullens and Luhring, personal observation).

In normal colony maintenance procedures, several dozen nematode males and females are placed together soon after emergence (Luhring and Mullens, 1997). They initially form tight, writhing clusters of nematodes, which then dissipate in the dishes of water after about 24 h, presumably after mating is complete (Mullens and Luhring, personal observation). Cluster mating was studied in the mosquito mermithid *S. spiculatus* by Dong et al. (2014), who showed that cluster mating enhanced mating success and was a result of females attracting both males and females.

Because this nematode-host system is amenable to laboratory maintenance and study, it provides several avenues of productive further research. First, it provides another possible system for theoretical study related to sex determination. Second, the host, *C. sonorensis*, is of considerable economic importance, and *H. magnapapula* is by far its most significant known natural enemy (Mullens and Luhring, 1998). While *H. magnapapula* does lack practical advantages, such as a resistant egg stage, which reduces potential for storage or transport compared with *Romanomermis* spp., it nevertheless deserves more study as a biological control agent. It seems not to disperse particularly well among habitats, for example, probably due to its very limited carryover into adult midges (Mullens and Velten, 1994; Paine and Mullens, 1994). One avenue for improved biological control, then, is the potential for human introduction of *H. magnapapula* into habitats where it does not yet exist. Further, *H. magnapapula* in the laboratory will attack and sometimes will develop in many other species of *Culicoides* besides *C. sonorensis* (Mullens et al., 1997). Thus, there might be situations where the nematode has potential against other important biting midge species.
